# What is the relationship between health-related quality of life among scoliosis patients and their caregiver burden? A cross-sectional study in China

**DOI:** 10.1186/s40359-023-01375-0

**Published:** 2023-10-19

**Authors:** Zhao Shi, Zhuxin Mao, Shiwen Xue, Gang Chen, Shunping Li

**Affiliations:** 1 Centre for Health Management and Policy Research, School of Public Health, Cheeloo College of Medicine, Shandong University, Jinan, 250012 China; 2 NHC Key Lab of Health Economics and Policy Research, Shandong University, Jinan, 250012 China; 3 Center for Health Preference Research, Shandong University, 250012, Jinan China; 4 Centre for Health Economics Research and Modelling Infectious Diseases (CHERMID), University of Antwerp, Antwerp, 2000 Belgium; 5 National Center for Respiratory Medicine, Guangzhou, 510000 China; 6 The First Affiliated Hospital of Guangzhou Medical University, Guangzhou, 510000 China; 7 National Clinical Research Center for Respiratory Disease, Guangzhou, 510000 China; 8 Guangzhou Institute of Respiratory Health, Guangzhou, 510000 China; 9 Centre for Health Economics, Monash Business School, Monash University, Melbourne, 3145 Australia

**Keywords:** Scoliosis, Health-related quality of life, Caregiver burden, China

## Abstract

**Background:**

Caregivers play a vital role in the recovery of scoliosis patients, but limited studies evaluate the caregivers’ HRQoL and burden in health care. This study aimed to explore the health-related quality of life (HRQoL) of scoliosis patients and their caregivers, and identify the factors influencing caregiver burden in Eastern China.

**Methods:**

This cross-sectional study was conducted from August 2018 to January 2019 at the Shandong Provincial Hospital, Jinan, China. The HRQoL of scoliosis patients was measured by the Scoliosis Research Society-22r (SRS-22r), five-level EQ-5D (EQ-5D-5L) and Child Health Utility 9D (CHU9D). The caregivers’ questionnaires consist of the EQ-5D-5L, WHO-five wellbeing index (WHO-5), 22-item Zarit Caregiver Burden Interview (ZBI-22) and Social Support Rating Scale (SSRS). Spearman correlation coefficients were used to estimate the relationship among caregivers’ burden, social support, HRQoL, and SWB. Cohen’s effect size (Cohen’s *d*) was used to assess the ZBI-22 total score between different groups. Multiple stepwise hierarchical linear regression models were conducted to assess the associated factors of caregiver burden.

**Results:**

There were 59 scoliosis patients and their caregivers (n = 59) included in the analysis. The mean health state utility of adolescent scoliosis patients (n = 39) was 0.718 (95%CI: 0.654, 0.782) based on CHU9D and adult scoliosis patients (n = 20) was 0.663 (95%CI: 0.471, 0.855) based on EQ-5D-5L. The mean health state utility of male scoliosis patients (0.792/0.667) was higher than females (0.681/0.662) based on CHU9D and EQ-5D-5L (*p* > 0.05), respectively. The ZBI-22 total score of scoliosis patients’ caregivers was 27.86 (SD: 20.59). Scoliosis patients’ HRQoL was significantly inversely correlated with caregiver burden, and the HRQoL and subjective wellbeing (SWB) of caregivers were moderately and inversely correlated with caregiver burden. The regression results showed that the patients’ age and caregivers’ SWB were key characteristics associated with caregiver burden.

**Conclusions:**

The caregiver burden of adolescent patients was higher than that of adult patients, and the satisfaction rate of adolescent scoliosis patients was higher than that of adult scoliosis patients. Improving the functional state of scoliosis patients and providing appropriate nursing practice education from health professionals would be necessary to effectively improve caregivers SWB and alleviate caregiver burden.

**Supplementary Information:**

The online version contains supplementary material available at 10.1186/s40359-023-01375-0.

## Background

Scoliosis is defined as a lateral curvature of the spine in the coronal plane of more than 10° [1]. Adolescent scoliosis is the most common spinal deformity, which affects 1–3% of children in the at-risk population of primary and middle school students [2–6]. The number of scoliosis cases among primary and middle school students exceeds 5 million in China, and scoliosis has become the third-largest disease after obesity and myopia that endanger to the health of Chinese children and adolescents [7, 8]. Adult scoliosis may lead to a high level of functional disability secondary to back pain, radicular pain, spinal claudication and neurologic dysfunction [9–11].

Health-related quality of life (HRQoL) is a multidimensional concept that represents the patient’s overall perception of the impact of an illness and its treatment, including the individual’s physical, psychological, and social aspects of life [12]. Scoliosis leads to physical deformity and functional impairment, which influences the patient’s HRQoL [13–15]. HRQoL has been widely used to assess scoliosis patients’ clinical outcomes and the impact of interventions [13, 16, 17]. The generic or disease-specific instruments, such as Medical Outcomes Study 36-Item Short-Form Health Survey (SF-36), Scoliosis Research Society-22 (SRS-22) and Oswestry Disability Index (ODI) [18–20], have been used to evaluate the scoliosis patient’s HRQoL in China. Preference-based measures of HRQoL are commonly used to generate health state utility values for the calculation of quality-adjusted life years (QALYs) in cost-utility analysis (CUA) [21]. The EQ-5D-3L and EQ-5D-5L have been used to evaluate idiopathic scoliosis and adolescent idiopathic scoliosis patients, respectively [22, 23]. Since health is a culturally grounded concept, people from different cultural backgrounds tend to have different understandings of HRQoL [24, 25], such as “weather adaption,” “spirit,” and “complexion” were unique health preferences for the Chinese population [25]. To the best of our knowledge, no studies evaluate the health state utility of scoliosis patients in Mainland China. Furthermore, previous literature showed that there are differences in HRQoL, psychosocial, appearance and functional domains between adult and adolescent scoliosis patients [15]. Recent studies have demonstrated differences in health state utility scores by gender and treatment [22, 23]. However, few studies compare the health state utility scores between adult and adolescent scoliosis.

Scoliosis can not only cause back pain, breathing problems, cosmetic deformities, self-image distortion, reduced vitality, impaired mental health and quality of life [10, 15, 26, 27], but can create financial burden, stress and anxiety in the caregiver [28–30]. For example, it is reported that parents of female adolescent idiopathic scoliosis patients overestimate stress levels related to their child’s body deformity [31]. In addition, although surgical treatment improved sitting balance and quality of life for neuromuscular scoliosis patients, it did not necessarily improve the parents’ quality of life [32]. It was observed that the monthly household income, social support, emotional stress and Cobb angle of patients are related to caregiver burden [33]. Limited studies have reported the relationship between scoliosis patient caregivers’ HRQoL and burden. Furthermore, in several different conditions researchers found that higher subjective wellbeing (SWB) and higher positive psychological functioning may reduce caregiver burden [34–37]. SWB is a psychological resource and potentiates positive emotion [38, 39], which may buffer distress and protect caregivers from experiencing burden [34, 37]. Given that caregivers play a vital role in the recovery of scoliosis patients [28, 29], it is important to explore the relationships between caregiver burden and SWB among scoliosis patient caregivers. Specifically, it will be critical to provide better support resources for the caregivers, thus improving the quality of care for the patients.

The first aim of this study was to evaluate the HRQoL of scoliosis patients and their caregivers, and then to explore the relationships among HRQoL, SWB and caregiver burden. The second aim was to identify the main factors influencing caregiver burden in Eastern China.

## Materials and methods

### Participants and produce

A cross-sectional study was conducted from August 2018 to January 2019 at the Shandong Provincial Hospital, Jinan, China. Informed consent was obtained before respondents participated in the survey. The participants completed the investigation in hospital wards, where face-to-face interviews were conducted by an investigator from Shandong University. The inclusion criteria for patients and caregivers were (1) the patient was diagnosed with scoliosis by clinical diagnosis, (2) the caregiver’s duration of care was more than 1 month, and (3) willingness of the respondent to give informed consent. The exclusion criteria were as follows: (1) the patient had any other comorbidities that could affect quality of life which was assessed by the clinician, (2) paid caregivers, (3) the patient and/or caregiver were cognitively impaired.

### Instruments

We divided patients into two age groups: adult scoliosis patients (age ≥ 18 years old) and adolescent scoliosis patients (age < 18 years old) in this study [10]. We used Scoliosis Research Society-22r (SRS-22r) as a disease-specific instrument to measure HRQoL of adult and adolescent scoliosis patients. Moreover, generic preference-based HRQoL instruments including the five-level EQ-5D (EQ-5D-5L) and Child Health Utility 9D (CHU9D) were used to evaluate the health state utility of adult and adolescent scoliosis patients, respectively. The caregivers’ questionnaires included the EQ-5D-5L, WHO-five wellbeing index (WHO-5), 22-item Zarit Caregiver Burden Interview (ZBI-22) and Social Support Rating Scale (SSRS). Socio-demographic information of the respondents was collected.

### Health-related quality of life (HRQoL)

#### Generic preference-based HRQoL for adults

The five-level EQ-5D (EQ-5D-5L) was used [40, 41]. It has five dimensions: mobility, self-care, usual activities, pain/discomfort, and anxiety/depression, with each dimension having five response levels: no problems, slight problems, moderate problems, severe problems, and unable to/extreme problems [42]. The Chinese version of the EQ-5D-5L descriptive system was adopted [43], and the Chinese-specific EQ-5D-5L value set was used to calculate health state utility in this study [44].

#### Generic preference-based HRQoL for children and adolescents

The Child Health Utility 9D (CHU9D) was adopted [45]. It has nine dimensions (worried, sad, pain, tired, annoyed, schoolwork/homework, sleep, daily routine and ability to join in activities), with five levels within each dimension [45]. The Chinese version of CHU9D has been proved a reliable and valid instrument to measure HRQoL for children and adolescents aged 8–17 [46]. The Chinese child and adolescent-specific value set was used in this study [47].

#### Disease-specific instrument

Scoliosis Research Society-22 (SRS-22) contains 22 items, which were divided into five domains covering function/activity, pain, self-image/appearance, mental health, and satisfaction with management [48, 49]. The function domain (item 18) was revised in 2006 (SRS-22r) [50]. The domain score and total score range from 1 to 5, with a higher score indicating better HRQoL [49]. The reliability and validity of the simplified Chinese version of SRS-22r have been verified [14, 18, 51].

### Subjective wellbeing (SWB)

The WHO-five wellbeing index (WHO-5) is a short and generic global rating scale measuring the SWB of the respondents over a 14-day period [52]. It contains 5 simple and positively phrased items with a 6-point scale, including cheerful and in good spirits, calm and relaxed, active and vigorous, fresh and rested, daily life has been filled with things that interest me. The raw score ranges from 0 to 25, with 0 representing the worst possible and 25 representing the best possible wellbeing. A total score below 13 indicates poor wellbeing and it is an indication for testing depression under ICD-10 [53]. The Chinese version of WHO-5 was translated in 2007 [53], which was used in this study.

### Caregiver burden

The 22-item Zarit Caregiver Burden Interview (ZBI-22) is a widely used measure of burden in caregivers [54, 55]. The ZBI-22 consists of 22 items and examines caregivers’ concerns of burden in the relationship, emotional wellbeing, social and family life, finances, and loss of control over one’s life [56, 57]. All items are scored on a 5-point scale ranging from 0 to 4. The total score is computed by summing the 22 items, which ranges from 0 to 88 with higher scores indicating a higher burden [58]. The Chinese version of the ZBI-22 has been verified as a reliable and valid instrument among Chinese caregivers [59, 60].

### Social support

The Social Support Rating Scale (SSRS) has been widely used in social support research in China [61–64]. The SSRS consists of 10 items and measures 3 dimensions: objective support (3 items), subjective support (4 items), and utilization of social support (3 items). The SSRS total score ranges from 12 to 66, and higher scores indicate greater social support [65]. Previous studies have verified the psychometric properties of SSRS in China [66, 67].

### Statistical analysis

Descriptive statistics were presented as mean (standard deviation, SD), 95% confidence interval (CI) or composition ratio (%). Normality test using Shapiro-Wilk W test and normal Q-Q plots. Comparison between two groups satisfying normal distribution and homogeneity of variance using independent-sample *t*-test, non-parametric tests using the Mann-Whitney U test or Kruskal-Wallis H test.

Spearman correlation coefficients were used to estimate the relationship among caregivers’ burden, social support, HRQoL and SWB. The strength of the correlation (*r*) was interpreted as follows: *r* > 0.7 indicates strong; 0.3 < *r* < 0.7 indicates moderate; *r* < 0.3 indicates weak [68]. Cohen’s effect size (Cohen’s *d*) was used to assess the ZBI-22 total score between different groups. Cohen’s cut-offs: Cohen’s *d* < 0.2 = small; 0.2  ≤ Cohen’s *d* < 0.5 = moderate; Cohen’s *d* ≥ 0.5 = strong, Cohen’s *d* ≥ 0.8 large [69].

Multiple stepwise hierarchical linear regression models were conducted to assess the associated factors with caregiver burden. Scoliosis patients’ characteristics (such as age group, gender, educational level, employment status, health insurance, and duration of scoliosis) and HRQoL were entered in step 1. Caregivers’ characteristics (such as age, gender, educational level, employment status, duration of caregiving, and the relationship with the patient) were entered in step 2. Given the SWB can be considered a psychological resource to reduce the caregiver burden [34, 37], the caregivers’ HRQoL and SWB were entered in step 3. Finally, caregivers’ social support was entered in step 4. The variance inflation factor (VIF) was applied to detect the presence of collinearity, with VIF > 5 indicating collinearity [70]. The variable entry criterion was set to less than 0.05 (*p* < 0.05) to enter the model. R^2^ and Adjusted R^2^ were used to evaluate the goodness of fit of the model.

All of the tests were two-tailed, and *p* < 0.05 was considered to be statistically significant. Statistical analyses were conducted using the SPSS version 20.0 and Stata version 14.1.

## Results

### Characteristics of scoliosis patients and caregivers

A total of 140 scoliosis patients and caregivers were interviewed. Among them, 2 patients and 1 caregiver didn’t complete the questionnaire; 19 patients and caregivers were further excluded due to which couldn’t achieve one-to-one matching. Finally, 59 pairs of scoliosis patients and their caregivers were included in this study, representing a total study sample size of 118 (84.3%).

Table 1 showed the characteristics of scoliosis patients and their caregivers. The average age of scoliosis patients was 19.6 (SD: 14.6) years and 66.1% were adolescent patients. More than half (62.7%) of the patients had a secondary school and above education. 88.1% of the patients were not employed by the time of the survey and 76.3% of patients’ duration of scoliosis were more than 12 months. The average age of caregivers was 41.8 (SD: 9.9) years and 50.8% of the caregivers were female. Most of the caregivers were parents (83.1%) and had a secondary school and above education (74.6%). More than half (59.3%) of the caregivers had been taking care of patients for more than 12 months.

**Table 1:** Characteristics of scoliosis patients and caregivers

Characteristics	Patients (N = 59) N (%)	Caregivers (N = 59) N (%)
Age, years (mean ± SD)	19.6 ± 14.6	41.8 ± 9.9
Patients’ age group		
Adolescent patients (age < 18 years old)	39 (66.1)	NA
Adult patients (age ≥ 18 years old)	20 (33.9)	NA
Gender		
Male	17 (28.8)	29 (49.2)
Female	42 (71.2)	30 (50.8)
Educational level		
Illiteracy or primary school	22 (37.3)	15 (25.4)
Secondary school and above	37 (62.7)	44 (74.6)
Employment status		
Yes	7 (11.9)	30 (50.8)
No	52 (88.1)	29 (49.2)
Patients’ health insurance		
New Cooperative Medical System	25 (42.4)	NA
Urban employee & Urban residence	12 (20.3)	NA
Family self-fund	22 (37.3)	NA
Duration of scoliosis, months		
< 12	14 (23.7)	NA
≥ 12	45 (76.3)	NA
Duration of caregiving, months		
< 12	NA	24 (40.7)
≥ 12	NA	35 (59.3)
Relationship with the patients		
Parents	NA	49 (83.1)
Spouse	NA	4 (6.8)
Son/daughter	NA	2 (3.4)
Others (e.g., uncle, sister)	NA	4 (6.8)
Subjective wellbeing		
WHO-5 scores ≥ 13	NA	33 (55.9)
WHO-5 scores < 13	NA	26 (44.1)

NA: not applicable; SD: standard deviation

### The HRQoL of scoliosis patients and caregivers

Table 2 showed the SRS-22r score of patients with scoliosis; among all dimensions, the self-image dimension had the lowest mean score. Moreover, the satisfaction with the management of adolescent patients was statistically and significantly higher than that of adult patients (*p* < 0.05).

**Table 2:** SRS-22r score of patients with scoliosis (Mean ± SD)

	Adolescent patients (N = 39)	Adult patients (N = 20)	*p* value
Function/activity	3.42 ± 0.96	3.71 ± 0.95	0.234 ^a^
Pain	4.06 ± 0.76	3.71 ± 0.95	0.187 ^a^
Self-image/appearance	3.24 ± 0.83	2.96 ± 0.92	0.248 ^b^
Mental health	3.59 ± 0.66	3.63 ± 0.81	0.974 ^a^
Satisfaction with management	4.00 ± 0.66	3.55 ± 0.86	**0.048** ^a^
Total score	3.61 ± 0.61	3.49 ± 0.73	0.493 ^b^

Bold values indicate the *p* < 0.05. a: Mann-Whitney U test; b: independent-sample *t* test; SD: standard deviation; SRS-22r: Scoliosis Research Society-22r

The CHU9D utility of adolescent scoliosis patients was 0.718 (95%CI: 0.654, 0.782), and the EQ-5D-5L utility of adult scoliosis patients was 0.663 (95%CI: 0.471, 0.855) (Table 3). The mean health state utility of male scoliosis patients (0.792/0.667) was higher than females (0.681/0.662) based on CHU9D/EQ-5D-5L (*p* > 0.05), respectively. The dimension responses for CHU9D and EQ-5D-5L were showed in Additional files 1 and 2. The proportion of adolescent scoliosis patients reporting being able to join in activities problems was the highest (71.8%), followed by tired (64.1%). The proportion of adult scoliosis patients reporting pain/discomfort problems was the highest (75.0%), followed by usual activities (55.0%).

**Table 3:** The HRQoL of scoliosis patients and caregivers

	Mean (SD)	95%CI	Maximum	Minimum
Adolescent patients (age < 18 years old)				
CHU9D utility	0.718 (0.198)	(0.654, 0.782)	1.000	0.205
Male	0.792 (0.159)	(0.696, 0.888)	1.000	0.467
Female	0.681 (0.208)	(0.597, 0.765)	0.956	0.205
Adult patients (age ≥ 18 years old)				
EQ-5D-5L utility	0.663 (0.410)	(0.471, 0.855)	1.000	-0.348
Male	0.667 (0.247)	(0.274, 1.000)	0.942	0.365
Female	0.662 (0.448)	(0.423, 0.901)	1.000	-0.348
Caregivers				
EQ-5D-5L utility	0.861 (0.209)	(0.806, 0.915)	1.000	-0.182

HRQoL: Health-related quality of life; SWB: Subjective wellbeing; EQ-5D-5L: Five-level EQ-5D; CHU9D: Child Health Utility 9D.

The mean health state utility of scoliosis patients’ caregivers was 0.861 (95%CI: 0.806, 0.915) based on EQ-5D-5L (Table 3). The proportion of caregivers reporting anxiety/depression problems was the highest (52.5%), and the least problematic dimension was self-care (16.9%).

### Caregivers burden and social support

The scoliosis patients’ caregiver ZBI-22 total score was 27.86 (SD: 20.59). As indicated in Table 4, the adolescent patients’ caregiver burden was higher than the adult patients’ caregiver (*p* < 0.05), and a strong effect size (*d* = 0.69) was seen in the patient age group. Moreover, the scoliosis patients’ educational level, caregivers’ educational level and SWB were found to be significantly associated with caregiver burden (*p* < 0.05) and has a strong (*d* = 0.60) to large effect size (*d* = 0.80) among the three groups.

**Table 4:** Caregivers’ burden and social support of different characteristics

Characteristics	ZBI-22 score Mean (SD)	*p* value	Effect size (*d*)	SSRS score Mean (SD)	*p* value
Panel A: Scoliosis patients’ characteristics					
Patient age group		**0.012** ^a^	0.69		0.873 ^a^
Adolescent patients (age < 18 years old)	32.46 (20.72)			33.49 (7.97)	
Adult patients (age ≥ 18 years old)	18.90 (17.55)			32.40 (6.81)	
Gender		0.541 ^a^	0.18		0.867 ^a^
Male	30.47 (20.65)			33.65 (8.22)	
Female	26.81 (20.72)			32.90 (7.36)	
Educational level		**0.005** ^a^	0.80		0.888 ^a^
Illiteracy or primary school	37.55 (21.01)			33.32 (7.36)	
Secondary school and above	22.11 (18.28)			33.00 (7.77)	
Employment status		0.252 ^a^	-0.43		0.757 ^a^
Yes	20.14 (20.99)			33.43 (4.43)	
No	28.90 (20.52)			33.08 (7.91)	
Patient health insurance		0.216 ^b^	NA		0.617 ^b^
New Cooperative Medical System	32.00 (18.46)			34.44 (7.79)	
Urban employee & Urban residence	23.58 (17.41)			31.08 (8.16)	
Family self-fund	22.50 (24.20)			32.73 (6.98)	
Duration of scoliosis, months		0.831 ^a^	-0.02		0.242 ^a^
< 12	27.50 (17.36)			35.36 (7.83)	
≥ 12	27.98 (21.67)			32.42 (7.42)	
Panel B: Caregivers’ characteristics					
Gender		0.481 ^a^	-0.21		0.710 ^a^
Male	25.62 (19.96)			32.97 (7.43)	
Female	30.03 (21.19)			33.27 (7.80)	
Educational level		**0.021** ^a^	0.74		0.125 ^a^
Illiteracy or primary school	38.80 (22.95)			30.67 (5.53)	
Secondary school and above	24.14 (18.56)			33.95 (8.02)	
Employment status		0.240 ^a^	-0.35		0.538 ^a^
Yes	24.30 (19.40)			32.57 (8.08)	
No	31.55 (21.47)			33.69 (7.08)	
Duration of caregiving, months		0.312 ^a^	-0.33		0.605 ^a^
< 12	23.83 (18.50)			32.67 (7.24)	
≥ 12	30.63 (21.74)			33.43 (7.86)	
Relationship with the patients		0.393 ^b^	NA		0.677 ^b^
Parents	29.57 (20.93)			33.73 (7.64)	
Spouse	27.50 (25.09)			32.25 (3.78)	
Son/daughter	19.00 (5.66)			28.50 (2.12)	
Others (e.g., uncle, sister)	11.75 (10.15)			28.75 (10.40)	
Subjective wellbeing		**0.011** ^a^	-0.60		0.183 ^a^
WHO-5 scores ≥ 13	22.61 (20.06)			34.33 (7.57)	
WHO-5 scores < 13	34.54 (19.64)			31.58 (7.40)	

Bold values indicate the *p* < 0.05; a: Mann-Whitney U test; b: Kruskal-Wallis H test; ZBI-22: 22-item Zarit caregiver burden interview; SSRS: Social support rating scale; SD: Standard deviation.

The social support rating scale (SSRS) total score of caregivers was 33.12 (SD: 7.56), the objective support score was 6.93 (SD: 2.34), the subjective support score was 19.80 (SD: 4.81), and the support utilization score was 6.47 (SD: 2.17). Table 4 showed that there were no statistically significant differences in the SSRS total score of scoliosis patients and caregivers with different characteristics.

### Relationships among the caregivers’ burden, social support, HRQoL and SWB

Table 5 showed correlation coefficients among the caregivers’ burden, social support, HRQoL, and SWB. All scoliosis patients’ function (*r*=-0.392, *p* < 0.01), SRS-22r total score (*r*=-0.259, *p* < 0.05) and patients’ health state utility (*r*=-0.373, *p* < 0.01) were significantly inverse correlated with caregiver’s burden. Compared to adolescents, pain (r=-0.478, *p* < 0.05) and mental health (r=-0.553, *P* < 0.01) were also significantly and inversely correlated with caregivers’ burden among adults (Additional file 3). Caregiver HRQoL (*r*=-0.554) and SWB (*r*=-0.473) (*p* < 0.01) were moderately and inversely correlated with caregiver burden, and the caregiver SSRS total score was weakly (*r* = 0.299) and positively correlated with caregiver SWB (*p* < 0.05) (Table 5). Heterogeneity was observed between caregivers of adults and adolescents, the absolute magnitudes of negative correlations between SWB and burden were consistently larger among adolescent patients (Additional file 3).

**Table 5:** Spearman’s correlation coefficients between the caregivers’ burden, social support, HRQoL and SWB

	Caregivers’ burden			Caregivers’ social support						
		ZBI-22 score		Objective support		Subjective support		Support utilization		Social Support Rating Scale score
Panel A: Scoliosis patients’ HRQoL										
SRS-22r, function		-0.392**	-0.108		0.052		0.105		0.052	
SRS-22r, pain		-0.250	0.071		0.094		0.190		0.162	
SRS-22r, self-image		-0.176	-0.006		0.172		0.204		0.173	
SRS-22r, mental health		-0.232	0.042		0.138		0.120		0.170	
SRS-22r, satisfaction		0.194	-0.020		0.117		0.059		0.102	
SRS-22r, total score		-0.259*	0.016		0.159		0.246		0.192	
Health state utility		-0.373**	0.031		0.006		0.037		0.029	
Panel B: Caregivers’ burden, HRQoL, SWB										
Caregivers’ burden										
ZBI-22 score		1.000	0.042		-0.095		-0.027		-0.055	
HRQoL										
EQ-5D-5 L utility		-0.554**	0.160		0.218		0.028		0.221	
SWB										
Cheerful and in good spirits		-0.386**	-0.114		0.321*		0.090		0.228	
Calm and relaxed		-0.463**	-0.171		0.219		0.041		0.136	
Active and vigorous		-0.462**	-0.053		0.301*		0.144		0.246	
Fresh and rested		-0.382**	-0.034		0.315*		0.055		0.241	
Daily life has been filled with things that interest me		-0.481**	0.118		0.551**		0.271*		0.501**	
WHO-5 total scores		-0.473**	-0.502		0.375**		0.130		0.299*	

HRQoL: Health-related quality of life; SWB: Subjective wellbeing; SRS-22r: Scoliosis Research Society-22r; WHO-5: WHO-five wellbeing index; EQ-5D-5 L: Five-level EQ-5D; ZBI-22: 22-item Zarit caregiver burden interview. *p < 0.05, **p < 0.01

**Table 6:** Factors associated with caregiver burden in the multiple stepwise hierarchical linear regression

Variables	Model 1				Model 2				Model 3				Model 4			
	β (SE)	Beta	*p *value		β (SE)	Beta	*p *value		β (SE)	Beta	*p* value		β (SE)	Beta	*p* value	
SRS-22r, function	-7.949 (2.654)	-0.369	0.004		-7.105 (2.597)	-0.330	0.008		-6.210 (2.533)	-0.288	0.017		-4.814 (2.489)	-0.223	0.058	
Patient age group (Ref: Adolescent patients)					-11.468 (5.195)	-0.266	0.031		-11.652 (5.008)	-0.270	0.024		-10.934 (4.800)	-0.254	0.027	
Caregivers’ educational level (Ref: Illiteracy or primary school)									-12.506 (5.449)	-0.267	0.026		-7.082 (5.659)	-0.151	0.216	
SWB, WHO-5 scores													-0.906 (0.368)	-0.306	0.017	
Intercept	55.808 (9.664)		< 0.001		56.727 (9.360)		< 0.001		62.969 (9.424)		< 0.001		66.508 (9.130)		< 0.001	
R^2^	0.136				0.205				0.275				0.348			
F	8.968		0.004		7.226		0.002		6.940		< 0.001		7.205		< 0.001	
Adjusted R^2^	0.121				0.177				0.235				0.300			
ΔR^2^	0.136				0.069				0.069				0.073			
ΔF	8.968		0.004		4.874		0.031		5.267		0.026		6.078		0.017	

Beta: standardized regression coefficient. SE: standard error; SRS-22r: Scoliosis Research Society-22r; SWB: Subjective wellbeing; WHO-5: WHO-five wellbeing index

### Factors associated with caregiver burden

Table 6 presented the factors associated with caregiver burden in the regression analyses. Model 1 explained 13.6% of the total variance of caregiver burden. Among patient HRQOL, function domain score (Beta=-0.369, *p* < 0.05) was the significant factor associated with caregiver burden. Model 2 showed that function domain score (Beta=-0.330, *p* < 0.05) and patient age group (Beta=-0.266, *p* < 0.05) together explained 20.5% of the total variance in caregiver burden. When further considering caregivers’ educational level, model 3 explained 27.5% of the total variance in caregiver burden. Model 4 accounted for 34.8% of the variance in caregiver burden with the effect of the patient age group (Beta=-0.254, *p* < 0.05), and WHO-5 scores (Beta=-0.306, *p* < 0.05) being the variables that contributed to the model.

## Discussion

This study evaluated the scoliosis patients’ HRQoL and their caregivers’ burden, HRQoL, SWB, and social support. To our knowledge, this is the first study to evaluate the health state utility of scoliosis patients in Mainland China. We found that HRQoL of scoliosis patients were inversely correlated with caregiver burden, and the HRQoL and SWB of caregivers were moderately and inversely correlated with caregiver burden.

The mean health state utility of male scoliosis patients (0.792/0.667) was higher than females (0.681/0.662) based on CHU9D/EQ-5D-5L (*p* > 0.05), respectively. The results were consistent with previous studies in Hongkong [22] and Sweden [23], which both demonstrated slightly higher scores for males. The adolescent scoliosis patients have lower HRQoL, which is lower than the SRS-22 norm scores of Chinese adolescents except for satisfaction with the management dimension [71]. Previous studies have demonstrated that adolescent scoliosis patients may experience psychosocial distress and body image issues, especially while undergoing treatment for scoliosis [15, 72]. Psychological intervention and physical exercise may help to ameliorate the potentially negative impact of scoliosis on their HRQoL [73, 74]. This study found that the satisfaction rate of adolescent scoliosis patients was higher than the adult scoliosis patients (*p* < 0.05). The adolescent scoliosis patients generally have good surgical results and associated decreased deformity, and they anticipate continued improvement. The Chinese government has implemented scoliosis screening incorporated into the routine physical examination of primary and secondary school students [7]. Early detection and diagnosis are the best prevention and treatment strategies for adolescent scoliosis patients, and it will improve their HRQoL.

The mean health state utility of caregivers was 0.861 (SD: 0.209) based on EQ-5D-5L and which was lower than the norm of the Chinese population (0.946, SD: 0.096) (*p* < 0.05) [75]. Furthermore, this study found that the caregiver burden was inversely and moderately correlated with their HRQoL (*r*=-0.554) (*p* < 0.01). The experience of scoliosis and spinal surgery for the caregiver, particularly the parent caregiver, can be physically exhausting, emotionally stressful and creates uncertainty [28, 29]. In addition, mothers experience frustration and disappointment with the lack of individualized information and support [28, 76]. Our findings also showed that burden for an adolescent patient’s caregiver was higher than the adult patient’s caregiver. This result was similar to previous studies, which reported the immense physical, psychological and financial burden on children and adolescent’s patient caregivers as they endured arduous rehabilitation periods, sleep deprivation and disability of their children [28, 29].

Regarding the relationship between caregiver burden and SWB of scoliosis caregivers, our study found that the caregiver SWB has a negative correlation with caregiver burden, and the caregiver with poor wellbeing (WHO-5 scores < 13) usually experiences a higher burden than the caregiver with high wellbeing (WHO-5 scores ≥ 13) (*p* < 0.05). The multiple stepwise hierarchical linear regression inclusion of WHO-5 scores (Model 4) contributed to the largest (7.3%) variance in caregiver burden score among all factors. These results suggest the caregivers SWB plays an important protective role in caregiver burden. The wellbeing may be part of a broader profile of psychosocial resilience and mediate coping strategies [38, 39]. Subjective and psychological wellbeing can be considered as psychological resources and positive emotions to be used when dealing with chronic illnesses and reducing caregiver burden [39, 77]. We found that scoliosis patients’ HRQoL was inversely correlated with caregiver burden, and the function domain (*p* = 0.058) appeared to be the associated factor with caregiver burden. The Cobb angle or body image usually affects satisfaction with treatment outcomes and caregiver burden. Furthermore, the previous findings highlighting a sense of confidence in dealing with the illness itself may result in a better sense of control and in the maintenance of caregivers wellbeing [35]. Healthcare professionals can help reduce caregiver burden by providing opportunities to develop caregiving skills through psychoeducational instruction, providing appropriate medical information and enhancing support resources [28, 29, 76].

There are several limitations to this study. Firstly, a relatively limited number of scoliosis patients and their caregivers were recruited from one hospital in Eastern China and the conclusions may not be representative of the whole scoliosis patients in Mainland China. While this was a relatively small sample, it did have the benefit of understanding the HRQoL of scoliosis patients and identifying the main factors influencing the caregiver burden, with efforts to support individuals and their careers. Secondly, limited clinical information, such as the specific classification of scoliosis, disease severity, presence of comorbidities, was collected in this study. Consequently, there might be other confounding factors that have not been accounted for in our analyses. Finally, the current cross-sectional study design limited its ability to draw causal inferences. Future research should consider a longitudinal study design to investigate the changes in HRQoL of scoliosis patients and caregiver burden over time.

## Conclusions

The adolescent patients’ caregiver burden was higher than the adult patients’ caregiver, and the satisfaction rate of adolescent scoliosis patients was higher than adult scoliosis patients. Poorer HRQoL was found for females than males. Improving the functional state of scoliosis patients and providing appropriate nursing practice education from health professionals would be necessary to effectively improve caregivers SWB and alleviate caregiver burden. It is clear that these patient-reported outcomes provide the clinician researcher with a vital outcome measure to evaluate our interventions and they should become a standard part of our patient evaluation.

### Electronic supplementary material

Below is the link to the electronic supplementary material.

Supplementary Material 1

## Data Availability

The datasets generated and/or analyzed during the current study are not publicly available due to anonymity of the participants but are available from the corresponding author on reasonable request.

## Abbreviations

CHU9D: Child Health Utility 9D
CI: Confidence interval
CUA: Cost-utility analysis
EQ-5D-5L: Five-level EQ-5D
HRQoL: Health-related quality of life
ODI: Oswestry Disability Index
QALYs: Quality-adjusted life years
SD: Standard deviation
SF-36: Medical Outcomes Study 36-Item Short-Form Health Survey
SSRS: Social Support Rating Scale
SWB: Subjective wellbeing
SRS-22: Scoliosis Research Society-22
SRS-22r: Scoliosis Research Society-22r
VIF: Variance inflation factor
WHO-5: WHO-five wellbeing index
ZBI-22: 22-item Zarit Caregiver Burden Interview
