# Supplementary material for: What is the relationship between health-related quality of life among scoliosis patients and their caregiver burden? A cross-sectional study in China
Source: BMC Psychol. 2023 Oct 19;11:346. doi: 10.1186/s40359-023-01375-0 (PMC10588232; doi:10.1186/s40359-023-01375-0)
Supplement: Supplementary file 1 — Supplementary Material 1 [file 40359_2023_1375_MOESM1_ESM.doc]

**What is the Relationship Between Health-related Quality of Life Among Scoliosis Patients and Their Caregiver Burden? A Cross-sectional Study in China**

Zhao Shi ^1, 2, 3^, Zhuxin Mao ^4^, Shiwen Xue ^5, 6, 7, 8^, Gang Chen ^9^ and Shunping Li ^1, 2, 3 *^

1. Centre for Health Management and Policy Research, School of Public Health, Cheeloo College of Medicine, Shandong University, 250012 Jinan, China
2. NHC Key Lab of Health Economics and Policy Research (Shandong University), 250012 Jinan, China
3. Center for Health Preference Research, Shandong University, 250012 Jinan, China
4. Centre for Health Economics Research and Modelling Infectious Diseases (CHERMID), University of Antwerp, 2000 Antwerp, Belgium
5. National Center for Respiratory Medicine, 510000 Guangzhou, China
6. The First Affiliated Hospital of Guangzhou Medical University, 510000 Guangzhou, China
7. National Clinical Research Center for Respiratory Disease, 510000 Guangzhou, China
8. Guangzhou Institute of Respiratory Health, 510000 Guangzhou, China
9. Centre for Health Economics, Monash Business School, Monash University, 3145 Melbourne, Australia

* Correspondence: [lishunping@sdu.edu.cn](mailto:lishunping@sdu.edu.cn)

**Additional files**


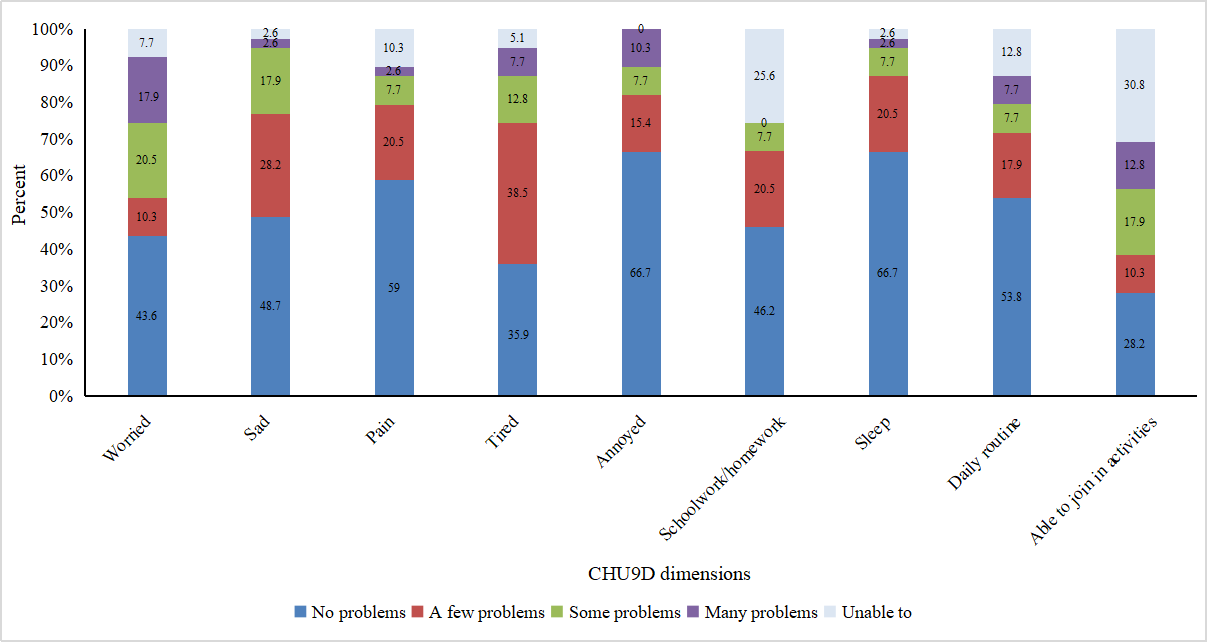


**Additional file 1 Distribution of CHU9D response levels of adolescent scoliosis patients (N=39)**


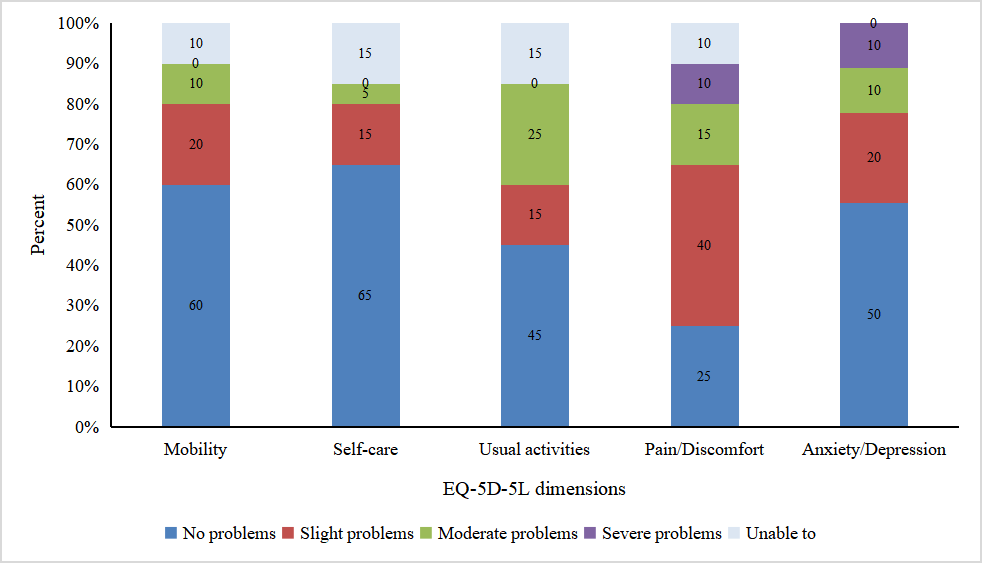


**Additional file 2 Distribution of EQ-5D-5L response levels of adult scoliosis patients (N=20)**

**Additional file 3 Spearman’s correlation coefficients between the caregivers’ burden, HRQoL and SWB**

|  | | Caregivers’ burden (ZBI-22 score ) | |
| --- | --- | --- | --- |
|  | Adolescent | | Adult |
| Panel A: Scoliosis patients’ HRQoL |  | |  |
| SRS-22r, function | -0.341* | | -0.478* |
| SRS-22r, pain | -0.281 | | -0.478* |
| SRS-22r, self-image | -0.278 | | -0.268 |
| SRS-22r, mental health | -0.107 | | -0.553* |
| SRS-22r, satisfaction | 0.030 | | 0.206 |
| SRS-22r, total scores | -0.303 | | -0.432 |
| Health state utility | -0.321* | | -0.509* |
| Panel B: Caregivers’ HRQoL, SWB |  | |  |
| HRQoL |  | |  |
| EQ-5D-5L utility | -0.616** | | -0.450* |
| SWB |  | |  |
| Cheerful and in good spirits | -0.433** | | -0.321 |
| Calm and relaxed | -0.479** | | -0.445* |
| Active and vigorous | -0.571** | | -0.420 |
| Fresh and rested | -0.511** | | -0.289 |
| Daily life has been filled with things that interest me | -0.520** | | -0.516* |
| WHO-5 total scores | -0.548** | | -0.410 |

HRQoL: Health-related quality of life; SWB: Subjective wellbeing; SRS-22r: Scoliosis Research Society-22r;

WHO-5: WHO-five wellbeing index; EQ-5D-5L: Five-level EQ-5D; ZBI-22: 22-item Zarit caregiver burden interview.

**p*<0.05, ***p*<0.01
